# Metastatic pancreatic cancer presenting with pulmonary arterial hypertension

**DOI:** 10.15537/smj.2023.44.2.20220632

**Published:** 2023-02

**Authors:** Abdulrahman H. Alharbi, Nora A. Alrajhi, Abdullah K. Alfaris, Muhannad R. Hawari

**Affiliations:** *From the Department of Medicine, King Faisal Specialist Hospital, Riyadh, Kingdom of Saudi Arabia.*

**Keywords:** pulmonary hypertension, chronic cough, metastasis, Group IV PH

## Abstract

Chronic cough is one of the most common symptoms encountered in respiratory clinics. It has a variety of differential diagnoses, and without a clear algorithmic approach, the final diagnosis can be overlooked. In this case report, we present a unique case of metastatic pancreatic cancer presented as chronic cough.

**R**ecurrent subacute pulmonary embolization leading to pulmonary arterial hypertension is an unusual presentation of malignancy.^1^ Tumor cells may reach the pulmonary vasculature in one of four ways: large, proximal emboli, generalized lymphatic dissemination (lymphangitic carcinomatosis), pure microvascular disease, or a mixture of these mechanisms.^2^ It is rare, underdiagnosed, and is usually recognized after a patient’s death. Here, we report the case of a patient with pancreatic cancer who presented with a cough which rapidly progressed to pulmonary hypertension (PH).

## Case Report

### Patient information

The patient was a 53-year-old male who was referred to the Pulmonary Medicine clinic for a dry cough for 6 weeks, associated with minimal sputum production which was yellowish in color without blood. The cough had no specific diurnal variation; it was not affected by the his position (supine or sitting), no aberrations during weekdays and weekends, and no definite aggravating or relieving factors (not affected by irritants or exercise). He did not complain of post-nasal drip or a history of the constant need to clear his throat. Additionally, there was no history of significant gastroesophageal reflux disease (GERD) symptoms or medication commonly associated with cough. A review of system showed remarkable night sweating and unintentional weight loss of 4 kg after noticing his symptoms.

His past medical history included ischemic heart disease, for which he underwent percutaneous coronary intervention (PCI) of the right coronary artery (RCA) in April 2021 (respiratory symptoms started 4 weeks after PCI) and dyslipidemia, with no significant past surgical history. He was taking ticagrelor, aspirin, atorvastatin, ezetimibe, and bisoprolol. There was no significant social history of exposure or occupation; he had an office job. He was an ex-smoker with a 10 pack-year history, and stopped at the age of 42. He had no relevant family history, apart from his mother, who had chronic lymphocytic leukemia. He had received a single dose of the COVID vaccine (AstraZeneca).

**Figure 1:**
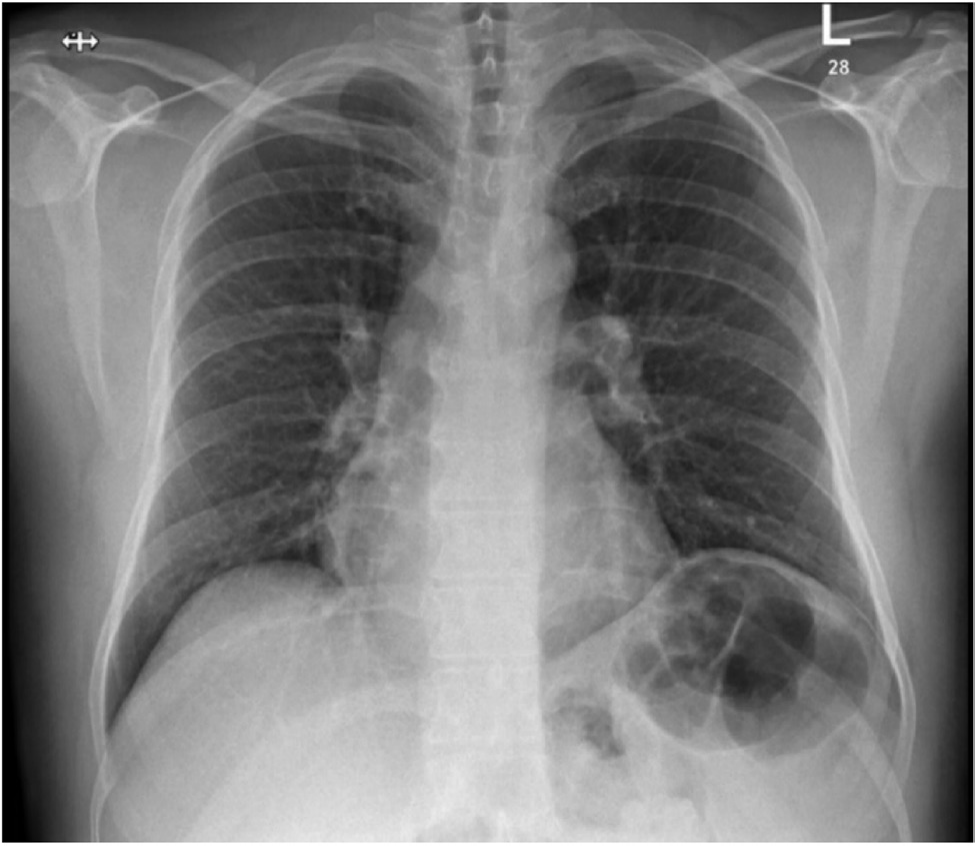
- Chest computed tomography. No focal active lung lesion is seen. The heart is not enlarged. Both cardiophrenic angles are clear.

Before he visited our clinic, he had consulted with the emergency departments and family medicine clinics, where he was treated empirically with inhalers (long-acting beta agonist, long-acting muscarinic antagonist, ICS), antibiotics, proton pump inhibitor, and a course of prednisone with no benefits. Chest radiography revealed no significant abnormalities (Figure 1).

### Clinical finding

Findings of physical examinations, including vital signs, ENT, chest, cardiovascular, lower limb, musculoskeletal, and neurological examinations were normal.

### Diagnostic assessment

Blood test results, including complete blood count, coagulation profile, renal profile, hepatic function, and electrolytes, were all normal. Laboratory workups for collagen vascular and autoimmune diseases yielded negative results. His pulmonary function test was consistent with a moderate obstructive pattern and significant reversibility (forced vital capacity [FVC]: 2.72 L [62%]; forced expiratory volume in 1 [FEV1]: 1.99 L [59%]; Post bronchodilator: 2.28 L, elevated by 14%; FEV1/FVC: 73%; residual volume [RV]: 101%; total lung capacity [TLC]: 80%; RV/TLC: 127%; and diffusion capacity for carbon monoxide: 105%). The microbiological workup results were negative for 3 acid-fast bacilli samples, sputum culture, Legionella, and respiratory multiplex for viral and atypical bacteria. Results for inflammatory markers were normal.

**Figure 2:**
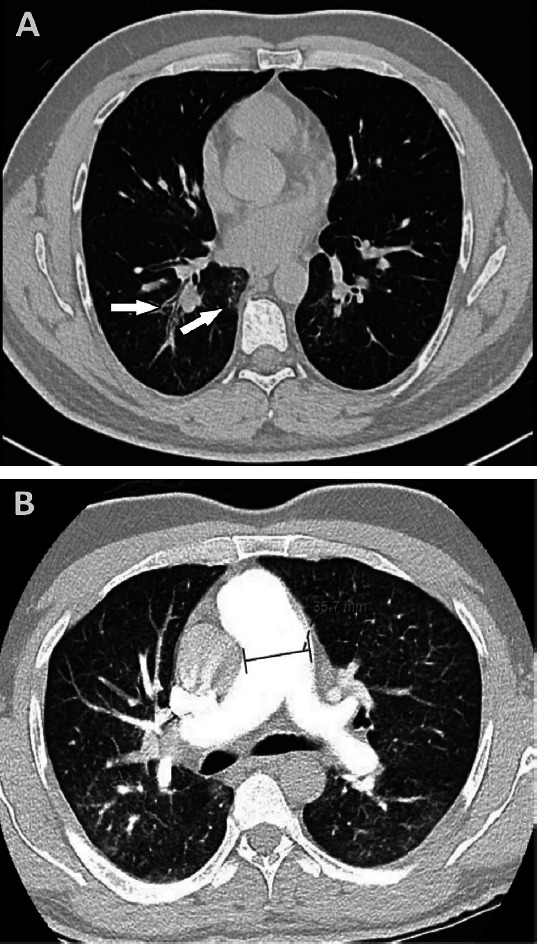
- High resolution computed tomography scan of the chest. (A) Bilateral faint ground glass opacities with tree-in-bud appearance and mild bronchial wall thickening, more pronounced at the right lower lobe; (B) The main pulmonary trunk is enlarged by 3.3 cm.

His initial chest CT showed bilateral faint ground glass opacities with a 3-in-bud appearance and mild bronchial wall thickening which was more pronounced in the right lower lobe, suggestive of an endobronchial infection (Figure 2). The echocardiographic results were normal. Ticagrelor was substituted with clopidogrel, as there have been reports that it can cause cough; however, the cough did not improve.

### Follow up and outcome

Two months later, the patient visited the clinic with a worsening cough to the point where it affected his daily activity and caused sleep disturbance. The patient was admitted for further assessment. Computed tomography sinuses were normal; he was also examined by an ENT specialist who found normal mobilizing vocal cords and no upper airway abnormalities. Bronchoscopy was attempted, but aborted as the patient desaturated and required 5 L/min oxygen administration. Hence, a CT - pulmonary embolism [PM] Study was performed as his D-dimer level was elevated, and was insignificant for PE. However, it showed bilateral ground glass opacities with septal thickening suggestive of edema and mildly enlarged right atrium with prominent right ventricle and straightening of the interventricular septum (Figure 2) in comparison to a previous CT. Repeated echocardiography showed moderate to severe tricuspid regurgitation and a right ventricular systolic pressure of 77 mmHg, while the systolic pressure on the left side was normal. His pro-natriuretic peptide (pro-brain natriuretic peptid) level was 3000 pg/mL. Hence, he was treated with an intravenous diuretic, which led to an improvement in his shortness of breath and normalization of oxygen saturation in the room air.

**Figure 3:**
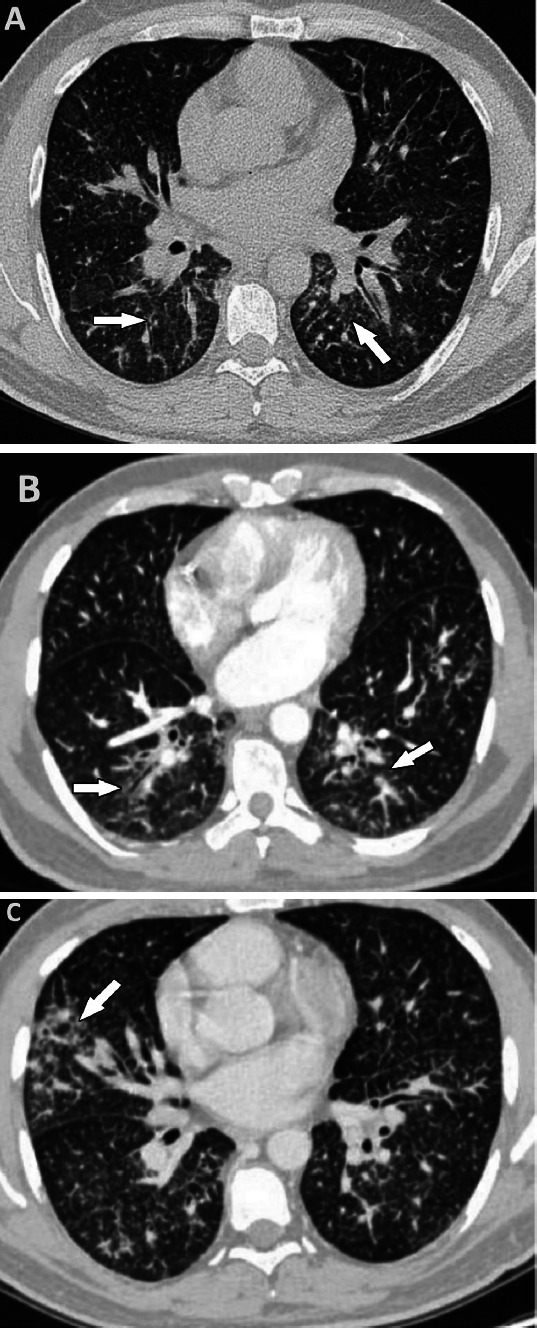
- Three High resolution computed tomography [HRCT] scan carried out one month apart showing progressive Ground Glass Opacities and nodular septal thickening. A) First HRCT. B) Second HRCT. and C) Third HRCT.

Right heart catheterization confirmed the presence of severe precapillary PH with an mean pulmonary artery pressure of 68 mmHg, PCW: 14 mmHg, Frick CI: 1.8 L/min/m2, Frick CO: 3.29 L/min, and PVR: 16.4 WU. He was evaluated by a PH team. The shortness of breath progressed, and he became oxygen-dependent; hence, he was initiated on macitentan (10 mg) under close observation. He showed some improvement in his shortness of breath but continued to have a persistent cough with no significant change in his oxygen requirements. Positron emission tomography (PET)-CT, performed due to excessive weight loss and persistent cough, showed normal findings. His cough and shortness of breath progressively worsened. His oxygen requirements increased, and he became oxygen-dependent and wheelchair-bound.

He later presented to the clinic with a 4-day history of neck swelling, which was suggestive of lymph node enlargement. He underwent ultrasound-guided fine-needle aspiration, which showed a metastatic adenocarcinoma with a pancreatic origin (serum CK7, CK19, and MUC-1 were positive). Magnetic resonance imaging abdomen showed a focal pancreatic neck mass, best seen in diffusion-weighted images and T1-weighted images—pre- and post-contrast, without pancreatic ductal dilatation, upper abdominal lymphadenopathy, or thoracic or lumbar spine metastasis. Hence, the final diagnosis was metastatic pancreatic carcinoma with a pulmonary tumor embolism and lymphangitic carcinomatosis.

Bone marrow biopsy was performed as the patient developed pancytopenia; it showed marked fibrosis with diffuse infiltration of the bone marrow tissue by clusters of malignant cells and a marked reduction in trilineage hematopoiesis. The patient was started on capecitabine with the dose reduced by 25% because of thrombocytopenia; however, his condition progressively worsened clinically and radiologically, with a further drop in platelets. Therefore, his code status was changed to “do not resuscitate” (DNR) and kept only on palliative treatment. Unfortunately, soon after, the patient passed away.

**Figure 4:**
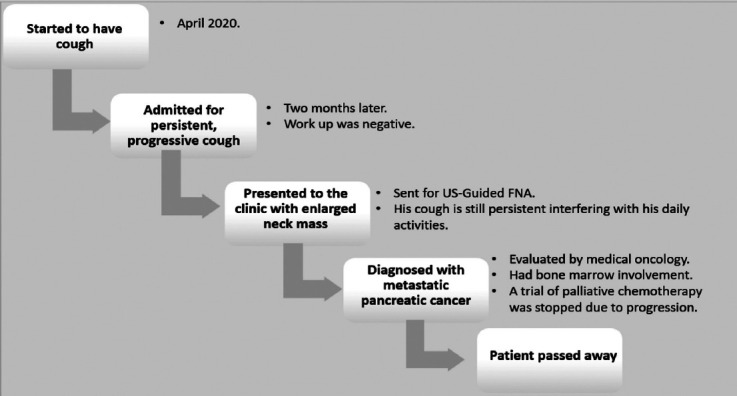
- Patient’s timeline.

## Discussion

Tumoral PH was recently classified as Group IV as a non-thromboembolic cause of PH via “other pulmonary artery obstruction”.^3^ The disease has a very poor prognosis, and most patients die a few weeks after diagnosis. Additionally, the diagnosis and treatment are challenging, and the majority of these cases are recognized after the diagnosis of a primary cancer.^4^ Most commonly, gastric cancer along with other cancers, such as pancreatic, lung, breast, colon, and urinary bladder cancers, have been associated with this condition.^5^ In this report, the patient was initially diagnosed with PH; right-sided heart catheterization with cytology is conventionally employed to establish the diagnosis of pulmonary tumor thrombotic microangiopathy. However, given our patient’s peculiar presentation and inconclusive results of initial imaging, mainly CT chest and PET-CT, it was not performed.

A similar case was reported in Saudi Arabia in a young woman who had respiratory symptoms and interstitial changes on CT imaging. A lung biopsy confirmed the presence of a moderately differentiated adenocarcinoma and immunohistochemistry suggested a GI origin of the neoplasm.^6^ Another patient with multiple recurrent upper and lower limb ischemia, required thrombectomy, direct thrombolysis, and revascularization surgery on different occasions during the same admission period and the tumor emboli was diagnosed after analyzing the last surgically removed embolus, which demonstrated a few clusters of malignant cells indicative of squamous cell carcinoma.^7^ However, both patients died soon after diagnosis, without confirming the underlying primary tumor.

A previous report on PH in Saudi Arabia assessed the demographics, clinical characteristics of patients, their treatment, and survival outcomes; 68 patients were categorized into group IV and all of them were diagnosed with CTEPH according to the data from the SAUDIPH registry. This was the first study to evaluate the data on pulmonary arterial hypertension from the SAUDIPH registry.^8^

In conclusion, this case demonstrate the challenges of diagnosing pulmonary tumor thrombotic microangiopathy despite advancements in diagnostic imaging and the associated high fatality, despite advances in oncological treatments, mostly due to the aggressive behavior of the underlying malignancy and the relatively late diagnosis. This case report can also aid our fellow doctors and practicing clinicians during the diagnosis of similar, rare cases.
